# Selecting antibacterial aptamers against the BamA protein in *Pseudomonas aeruginosa* by incorporating genetic algorithm to optimise computational screening method

**DOI:** 10.1038/s41598-023-34643-5

**Published:** 2023-05-10

**Authors:** Rupany Selvam, Ian Han Yan Lim, Jovita Catherine Lewis, Chern Hong Lim, Michelle Khai Khun Yap, Hock Siew Tan

**Affiliations:** 1 School of Science, Monash University Malaysia, Bandar Sunway, Selangor Malaysia; 2 School of Information Technology, Monash University Malaysia, Bandar Sunway, Selangor Malaysia; 3 Tropical Medicine and Biology Multidisciplinary Platform, Monash University Malaysia, Bandar Sunway, Malaysia

**Keywords:** Biotechnology, Computational biology and bioinformatics, Microbiology

## Abstract

Antibiotic resistance is one of the biggest threats to global health resulting in an increasing number of people suffering from severe illnesses or dying due to infections that were once easily curable with antibiotics. *Pseudomonas aeruginosa* is a major pathogen that has rapidly developed antibiotic resistance and WHO has categorised this pathogen under the critical list. DNA aptamers can act as a potential candidate for novel antimicrobial agents. In this study, we demonstrated that an existing aptamer is able to affect the growth of *P. aeruginosa.* A computational screen for aptamers that could bind to a well-conserved and essential outer membrane protein, BamA in Gram-negative bacteria was conducted. Molecular docking of about 100 functional DNA aptamers with BamA protein was performed via both local and global docking approaches. Additionally, genetic algorithm analysis was carried out to rank the aptamers based on their binding affinity. The top hits of aptamers with good binding to BamA protein were synthesised to investigate their in vitro antibacterial activity. Among all aptamers, Apt31, which is known to bind to an antitumor, Daunomycin, exhibited the highest HADDOCK score and resulted in a significant (p < 0.05) reduction in *P. aeruginosa* growth. Apt31 also induced membrane disruption that resulted in DNA leakage. Hence, computational screening may result in the identification of aptamers that bind to the desired active site with high affinity.

## Introduction

*Pseudomonas aeruginosa* is a Gram-negative opportunistic bacterium that causes many nosocomial infections, and it is a major pathogen in the cystic fibrosis lungs of infected patients. Besides, it is also associated with other hospital-acquired infections such as ventilator-associated pneumonia, urinary catheter-related infection, surgical/transplantation infection and many more^1^. Recently, it was also found that *P. aeruginosa* is the second most frequently detected pathogen in COVID-19 patients causing coinfection^2–4^. The World Health Organisation (WHO) has classified this deadly organism as an ESKAPE pathogen, and it is also categorised under the Priority 1: Critical list due to the emergence of multidrug-resistant (MDR) strains. Infections caused by *P. aeruginosa* are becoming more difficult and almost impossible to treat where certain strains developed resistance to almost all the currently available antibiotics, even to the last resort treatment, thereby increasing the morbidity and mortality rate worldwide^5^. Therefore, there is an urgent need to discover novel antimicrobial agents to overcome this global health crisis.

Production of antibiotics is costly and laborious where it takes almost 10 years for an antibiotic to reach the market, but it can take as early as 11 days for bacteria to develop resistance to it^6^. Hence, an alternative approach should be considered to prevent global poverty caused by antimicrobial resistance (AMR). Drug repurposing, which is also known as drug repositioning or drug reprofiling, has recently received enormous attention in the pharmaceutical industry as discovering new therapeutic indications of already existing drugs can reduce the time frame and cost of drug development. The safety and toxicity of repurposed drugs have also been well studied, and therefore can be considered safe for new therapeutic use. Hence, repurposing aptamers can reduce the cost and length of new aptamer discovery and drug development. Aptamers have emerged as promising tools for diagnostics and therapeutics in the microbiology field. Aptamers are folded short single-stranded oligonucleotides (DNA/RNA) that can specifically bind and form complexes with molecular targets such as proteins^7^. This binding may elicit therapeutic effects and many therapeutic aptamers are already undergoing clinical trials. For example, Macugen is an aptamer approved by FDA and available in the market as a drug to treat macular degeneration^7^. Aptamers are potential antimicrobial agents as it is cost-effective and most importantly, it possesses various advantages over antibodies and antibiotics such as low immunogenicity, short production timeline, and high stability^8^. Aptamers are also flexible for structural and chemical modifications, eventually extending their clinical applications^8^. Additionally, the identification of suitable targets in bacteria for aptamer binding is also important because the low permeability of the outer membrane layer in Gram-negative bacteria is one of the major reasons for AMR development^9^. Thus, the chosen target should be easily accessible without the need to pass through the low permeable outer membrane layer in *P. aeruginosa*, and therefore outer membrane proteins can be the potential targets.

BamA is a component of the β-barrel assembly machinery (BAM) complex, a complex that inserts β-barrel proteins into the outer membrane. It was found that the BamA is the core component of the BAM complex and it is highly conserved in Gram-negative bacterial species as well as it is exposed to the extracellular region^10^. BamA is vital for cell viability and bacterial growth whereby its inhibition exhibited antibacterial activity in many Gram-negative bacteria^11^. Formation of the lateral gate by β-strand 1 (β1) and β-strand 16 (β16) in BamA is important for the binding of nascent outer membrane proteins to BamA^12^. Nascent proteins enter the lateral gate, get folded and assembled, and exit via the pore of BamA. Therefore, targeting the lateral gate in BamA may inhibit bacterial growth and survival. Darobactin is an antibiotic isolated from *Photorhabdus* bacteria that can effectively kill many Gram-negative bacteria by inhibiting BamA activity^13,14^. Unfortunately, no study has explored the binding of aptamers to BamA to inhibit bacterial growth. We hypothesised that binding of aptamers to the lateral gate in BamA protein will prevent the assembly and insertion of β-barrel proteins into the outer membrane, thereby resulting in cell death.

Aptamers are usually selected through a long and laborious in vitro screening method known as Systematic Evolution of Ligands by EXponential enrichment (SELEX). Computational drug screening approach is gaining popularity among researchers because it is time-saving, cost-effective and reliable. Many studies have shown that in silico SELEX methods could efficiently drive aptamer design and selection to new heights besides being time-saving and cost-effective^15–17^. This lines up with the increasing attention received by in silico bioinformatic methods, as various approaches have been proposed, attempted, and tested for computational design and selection of aptamers whilst loaning many benefits. Molecular docking is often employed to characterise and simulate the binding affinities and structural properties of complexes, a common practice prior to in vitro experiments, thus saving time and labour. Hence, drug design studies favour docking simulations, and several algorithms have been developed since, such as HADDOCK, ZDOCK, HDock, AutoDock, AutoDock Vina, Rosetta and many more^18,19^. However, a few studies have advised that further work is required to analyse the binding complexes, despite finding their respective best structure^20^. This is due to varying binding scores between different docking software, including some possessing scores unable to be taken as the ‘true binding affinity’ as some are not calibrated to experimental data.

To date, many proposed methods of computational analysis of the structure and properties of aptamers have shown their effectiveness. Thus, to minimise errors in this field, more powerful and efficient software and algorithms will always be relevant, notably machine learning and artificial intelligence techniques which have been under recent interest. The application of machine learning and deep learning techniques for aptamer discovery and selection has begun to evolve^21–23^. Together, these factors necessitate additional case studies, experiments, and subsequently systematic and up-to-date reviews^19^.

Stochastic search algorithms that are often used in Machine learning applications such as genetic algorithms (GA) may offer a solution to some challenges in computational screening methods. GAs refer to mathematical algorithms which were inspired by Charles Darwin’s idea of natural selection, whereby the algorithm only preserves the fittest individuals over the different generations^24^. One of GA’s purposes was to use a number of variables or vectors (or *genes* in GA terms) to create a prediction model and find close-to-exact or approximate solutions for optimization and search problems^25^. The concept of GA is by combining different values every generation to extract the best combination of values in each variable to create novel individuals with the highest fitness^24^. For this study’s purposes, instead of using GA to utilise sequence and structural data in searching for the best-performing aptamer, we will use features operationalized into vectors, feeding GA in obtaining candidate aptamers.

Hence, in this study, we applied an in silico approach to select aptamers from an existing dataset, which consists of functional published DNA aptamers that are known to bind to various targets, that also bind to BamA protein in *P. aeruginosa* based on molecular docking and genetic algorithms analysis. We further determined its antibacterial activity via in vitro assays. The main objective of this study is to repurpose available aptamers as novel antibacterial agents by targeting the essential outer membrane protein through cost-effective and less laborious methods.

## Materials and methodology

### Construction and validation of universal *Pseudomonas* BamA model using homology modelling

To date, no structural model of *Pseudomonas* species BamA is available. Although BamA protein is conserved among Gram-negative bacteria species, *Pseudomonas* has a larger genetic distance than other bacteria such as *Escherichia coli*, *Salmonella and Shigella*^26,27^*.* Therefore, a universal *Pseudomonas* BamA model was constructed. Multiple sequence alignment was performed using COBALT (https://www.ncbi.nlm.nih.gov/tools/cobalt/re_cobalt.cgi) with all the 100 available *Pseudomonas* BamA amino acid sequences from 37 species (retrieved 20 March 2022). A consensus sequence of BamA was then derived. Modeller 10v1 software (https://salilab.org/modeller/) was used to construct the model based on a few templates from PDB (5d0oA, 6lyqA and 5aywA). The templates were chosen based on the highest percentage similarity of amino acid sequences with the consensus *Pseudomonas* BamA sequence. Discrete Optimised Protein Energy (DOPE) score profile, RMSD value, Ramachandran plot and PROCHECK analysis were used to validate the constructed *Pseudomonas* BamA model. Molecular docking between the *Pseudomonas* BamA model and Darobactin, the known BamA inhibitor, was carried out using HADDOCK 2.4 web server (https://wenmr.science.uu.nl/haddock2.4/) to determine its putative binding site. The docked model was compared with the available experimental Darobactin-bound BamA model from the PDB database (7P1C).

### Prediction of aptamer structures

Functional DNA aptamer sequences with no modification in neither the bases nor the backbone were obtained from the ‘Heredia F. DNA/Aptamer’ (https://data.mendeley.com/datasets/76jgjbgndr/1) dataset^28,29^. This data was previously web-scraped from the online database Aptagen using python script^28^. DNA aptamers with no modifications were selected because DNA is more stable compared to RNA and unmodified aptamers can be easily synthesised at a lower cost compared to modified aptamers. Out of the 238 aptamer sequences, about 100 aptamers were used for docking with the *Pseudomonas* BamA model. These 100 aptamer sequences were selected based on multiple criteria such as length (20–100 base pairs) and absence of degeneracy and modifications in the sequences. The ideal length of aptamers should be around 20–100 base pairs. Short aptamers may not fold into a defined structure, whereas long aptamers may not be able to penetrate through the bacteria cells. Thus, only aptamers with 20–100 base pairs were used for docking.The secondary and tertiary structures of selected aptamers were predicted using UNAFold Web Server (http://www.unafold.org/mfold/applications/dna-folding-form.php) and RNAComposer (https://rnacomposer.cs.put.poznan.pl/), respectively^30^.

### Molecular docking between aptamers and BamA protein using local and global docking approach

Molecular docking between the 3D structure of the aptamers and *Pseudomona*s BamA model was carried out using the HADDOCK 2.4 web server (https://wenmr.science.uu.nl/haddock2.4/) which utilises a local docking method whereby the active and binding sites of the ligands need to be specified^31^. Residues at the lateral gate of BamA (E401, S402, G403, S404, I405, T406, A407, S408, V409, G410, F411, E786, T787, Q788, V789, F790, Q791, F792, S793, L794, G795) were chosen as the active residues in our docking. The docking complexes with negative HADDOCK scores are considered more stable complexes while a positive HADDOCK score is considered less stable, and the binding may be energetically unfavourable. The aptamer candidates with the most negative (low) HADDOCK score were shortlisted for downstream analysis. Complexes with less negative (high) HADDOCK score were generated using random oligonucleotide sequence for molecular docking with the BamA. As such, the random oligonucleotide, HTO008, was chosen as the negative control based on the length, which is about 38 base pairs. Finally, the Residue Interaction Network Generator (RING) (https://ring.biocomputingup.it/submit)^32^ analysis was carried out to identify all types of non-covalent interactions at atomic levels in the aptamer-BamA complexes obtained from HADDOCK 2.4 web server, to demonstrate how strong the aptamers can bind to the lateral gate region of BamA. In addition, global docking was also carried out to determine the most favourable binding sites of the aptamers and the negative control in BamA. HDOCK server (http://hdock.phys.hust.edu.cn/)^33^ was used for the global docking approach without specifying the active sites. The docking in the HDOCK server is based on a hybrid algorithm of template-based modelling and ab initio free docking. Global docking results were compared with the local docking models, and the genetic algorithm ranking and antibacterial assay were analysed based on that. Based on the local and global docking analysis, the best aptamer with the most negative HADDOCK score that binds to the lateral gate region was selected.

### Genetic algorithm

#### Data cleaning

To potentially study appropriate features of good aptamer-BamA binding, the HADDOCK scoring output of 100 docked aptamers against BamA was extracted, and the five HADDOCK features or vectors were HADDOCK score, RMSD, Z-score, Van der Waals forces and electrostatic energy. This was conducted by sorting and statistically reporting distributions of each HADDOCK vector by mean and upper-lower limit.

#### Gaussian distribution modelling of HADDOCK vectors

To provide input data for a genetic algorithm, the vectors were normalised based on % from 0 to 100% due to varying scores, values, and units across the five vectors. For example, HADDOCK score range was − 130 to + 60, whilst electrostatic energy was − 676 to − 25. The HADDOCK output data of the 100 aptamers presents the five vectors influencing how well a binding aptamer performs with the BamA protein. In order to maximise our chances of finding the best combination of aptamer-BamA binding vectors, that would be present in a well-performing aptamer, we utilise a genetic algorithm to assist in that search. Thus, applying a genetic algorithm with the best aptamer vector data to solve the maximisation or optimisation problem, would require maximum vector scores from the best aptamers, which were classified based on the most-negative scores in HADDOCK and RMSD alone. However, amongst the vectors, the HADDOCK score was noted to only have less random distributed values, based on visual inspection of the parallel coordinates graph. As the other four vectors had the best aptamers at a more random and distributed spread across their score ranges, it would be more difficult and riskier to assume the most negative scores as attributes of best-performing aptamers in aptamer-BamA interaction.

It was noted that there were no standard scores for any of the five vectors, hence a Gaussian assumption was assumed for each vector. In addition, a multi-modal approach could be adopted to assign more than one Gaussian model for each of the other four vectors (excluding the HADDOCK score). The maximum value from the results of the multi-modal calculations based on the Gaussian membership function was used to build the fitness function.

$$ f\left( {x;\sigma ,c} \right) = e^{{\frac{{ - \left( {x - c} \right)^{2} }}{{2\sigma^{2} }}}} . $$

Gaussian membership function, whereby the x = input value, σ = standard deviation, c = mean.

#### Running GA: obtaining best distribution of HADDOCK vectors

Implementing a GA requires an equation to quantify the goal the algorithm is attempting to achieve. To operationalize the optimization goal this GA will achieve, the Gaussian model was employed to quantify our goal in obtaining the best distribution of scores in our five vectors: HADDOCK score (A1), RMSD (A2), Van der Waals forces (A3), electrostatic energy (A4), and Z-score (A5). Generally, a genetic algorithm requires five elements: its initial population, a fitness function, selection, mutation, and crossover operators.

##### Initial population

Essentially, an initial population consists of random values and scores of the selected features. For this study, random values from a scale of 0–100% were included, as the five vectors were normalised into percentages. In each vector, the five random numbers represent the genes in a chromosome (or array of random numbers), of which the ‘selected parents’ and ‘subsequent offspring’ are generated by applying the crossover and mutation operations. An example of a vector is as follows: [2.58, 1.886, 39.64, 60.94, 21.053].

##### Fitness function

A fitness function is defined simply as the objective function to be optimised. It is built by summation of each value in a vector, whereby each value is modelled through the respective Gaussian membership function. Thus, for the fitness function to generate new solutions based on existing HADDOCK vector data, the best solutions should reach a goal value of 5. Y (solution) = A1 + A2 + A3 + A4 + A5. The five vectors would be coded as A1 (HADDOCK score), A2 (RMSD), A3 (Van der Waals), A4 (electrostatic energy), and A5 (z-score) respectively. Using the Gaussian function, the GA would take the maximum of possible outputs from each vector and its Gaussian function and subsequently sum them together to get a number close to the optimal result of 5.

##### Selection, crossover, mutation

Genetic algorithms have functions and commands to improve their search for the best solution, known as parameters. The parameters typically used are as follows: num_generations = 500 (total number of populations in a single run), sol_per_pop = 100 (solution to generate in each population), parent_selecton_type = random (type of selecting parents in each generation), keep_parents = 1 (number of parents to keep in the current population), crossover type = single_point (type of crossover operation), mutation_type = random (type of mutation), mutation_num_genes = 1 (number of genes mutated per generation).

##### Ranking of aptamers based on Euclidean distance

Based on the best distribution of HADDOCK vectors, the Euclidean distance formula (√Σ(Ai-Bi)2) was utilised to rank and list the 100 aptamers in Excel, to observe how close the aptamers were to the ‘best distribution’. The Excel code is as follows: = SQRT(SUMXMY2(‘range of rows of the best distribution’, ‘range of rows of the specific aptamer’s vector distribution)). ‘SUMXMY2’ takes the sum of the squared differences in the corresponding elements of the two ranges, Ai and Bi. ‘SQRT’ finds the square root of SUMXMY2. The end result is the Euclidean distance between the two ranges. The smaller the distance, the closer the aptamer HADDOCK vectors are close to the best distribution for good aptamer-BamA binding.

### Antibacterial assay

Bacterial strain, *Pseudomonas aeruginosa* (ATCC 10145) was used as the target organism. Selected aptamer sequences were synthesised by Integrated DNA Technologies company. Luria–Bertani (LB) media, Nutrient Agar (NA) and Phosphate-Buffered Saline (PBS) were purchased from the HI-MEDIA brand.

#### Colony forming unit analysis of *P. aeruginosa* after aptamer treatment

After selecting the best binding aptamers, in vitro antibacterial assay was carried out. 100 μl of 20 μM aptamer in 1X Phosphate-buffered saline (PBS) (pH ~ 7.4) was denatured and renatured for tertiary structure folding by heating at 95 °C for 10 min followed by slow cooling using a thermocycler. An overnight *P. aeruginosa* culture with an optical density (O.D.) of 1 at 600 nm was collected and the cell pellet was washed with 1× PBS. After washing, the cells were centrifuged at 13,000 rpm for 5 min. The cell pellet was then resuspended with 100 μl of PBS followed by the addition of 100 μl of the folded aptamer to obtain 10 μM of aptamer as the final concentration. 100 μl of 1× PBS was added to the control (no treatment) group. For 0-h incubation, serial dilution was carried out immediately after adding aptamers using a multichannel pipette and 8 μl of cells from dilution 10^4^ to 10^6^ were spotted on the NA plate. Then, the bacteria were incubated with the aptamers for 1 and 2 h at 4 °C to slow the bacterial growth and allow binding. Spot plating was carried out for each time point and the plates were incubated at 25 °C overnight followed by 37 °C for 5 h. The colony forming unit (CFU/mL) for each aptamer condition was analysed and the experiment was repeated three times independently. The CFU/mL and percentage of bacterial growth were presented as bar charts.

#### DNA leakage in *P. aeruginosa* after aptamer treatment

Bacteria release their cytoplasmic contents, especially DNA, due to loss of membrane integrity. After incubating the bacteria under different treatment conditions for 2 h, the bacteria from each treatment condition were centrifuged at 13,000 rpm for 5 min and the supernatants were collected. The supernatants were then purified using FavorPrep GEL/PCR Purification Kit to remove the aptamers which are two times smaller than the cut-off base pair size of the membrane in the column. After purification, the supernatants should only contain the genomic DNA released by the cells. The DNA concentration was measured at 260 nm using the BioDrop µLITE machine and the concentration of DNA released in the presence of aptamers was compared with the control (no treatment) group to study the membrane integrity.

### Statistical analysis

At least three experimental replicates were carried out for all assays. All the data are presented as the means ± standard deviations. Bartlett’s test was carried out to assess the equality of variance in different groups, and ordinary One-way ANOVA with multiple comparisons was carried out to compare the mean values between control and treatment groups using GraphPad Prism. The significance level was set at *p* < 0.05.

## Results

### Universal *Pseudomonas* BamA model

**Figure 1:**
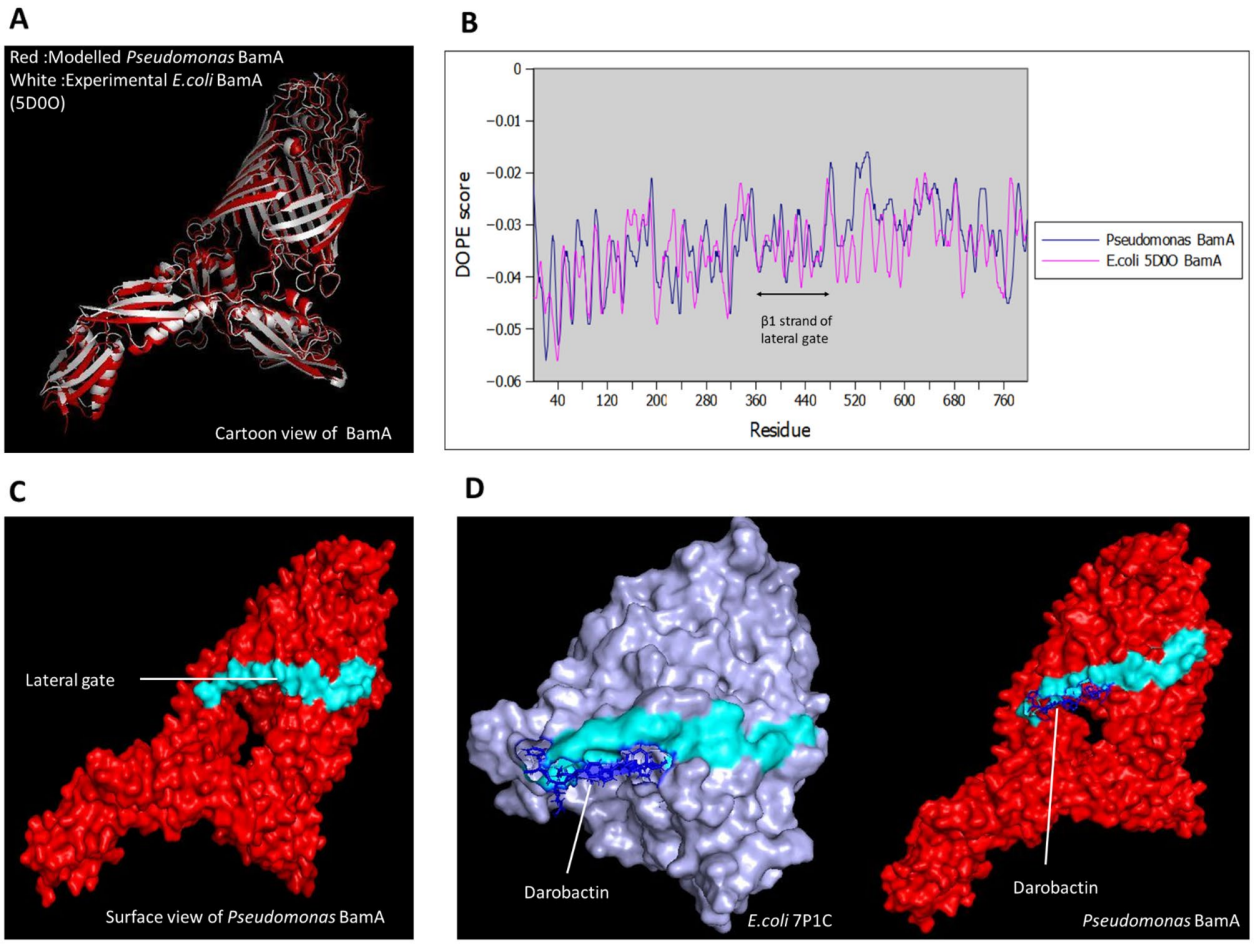
Structural modelling of *Pseudomonas* BamA. (**A**) Superimposition of modelled *Pseudomonas* BamA (red) and 5D00 *E. coli* BamA (white) resulting in RMSD value of 1.781 Å. (**B**) Plots of DOPE scores of *Pseudomonas* BamA and 5D0O *E. coli* BamA showing conserved β1 strand in lateral gate. (**C**) Final *Pseudomonas* BamA model and the lateral gate is highlighted (cyan). (**D**) *Pseudomonas* BamA (Red) and 7P1C (Light purple) show bound Darobactin (Blue) to the lateral gate.

Due to the unavailability of BamA model for *Pseudomonas* species, a universal *Pseudomonas* BamA model was built. Multiple sequences alignment of BamA protein from around 37 strains showed that *P. aeruginosa* shared about 82% similarity in the amino acid sequence with other *Pseudomonas* species (Supplementary Fig. 1), which indicates that the BamA is conserved among the *Pseudomonas* species. The consensus sequence obtained (Supplementary Fig. 1) was used as the target sequence for BamA modelling. Since BamA protein is highly conserved among Gram-negative species, the modelled BamA protein was superimposed with the experimentally determined PDB structure of BamA from *E. coli* (5D0O) to determine the structural similarity. The RMSD value of the modelled BamA was 1.781 Å (Fig. 1A), which suggests that modelled BamA was structurally similar to the experimental *E. coli* BamA (5D0O). Furthermore, the DOPE score profiles of both *E. coli* (5D0O) and modelled *Pseudomonas* BamA were plotted and compared (Fig. 1B). Although the DOPE score profiles of the models appear quite different from each other due to the variation in amino acid sequences, they were similar between amino acid residues 360–480 which includes the lateral gate region (residues 400–410). Besides, the modelled BamA was also validated with PROCHECK analysis (Supplementary Fig. 2). The finding showed that the modelled BamA protein was within the acceptable criteria where > 90% of the residues fell under the most favoured region. Besides, the Ramachandran plot (Supplementary Fig. 2) also predicted that the model consisted mostly of β-sheets with small segments of *α*-helix which corresponded to the secondary structures BamA and other outer membrane proteins. The final *Pseudomonas* BamA structure with a lateral gate is shown in Fig. 1C. Darobactin was docked with *Pseudomonas* BamA to determine its binding site. The experimental PDB structure of *E. coli* (7P1C) and modelled *Pseudomonas* BamA bound to Darobactin were shown for comparison in Fig. 1D. The similar binding site in both experimental and modelled structures further validates the *Pseudomonas* BamA model.

### Molecular docking between aptamers and BamA

**Figure 2:**
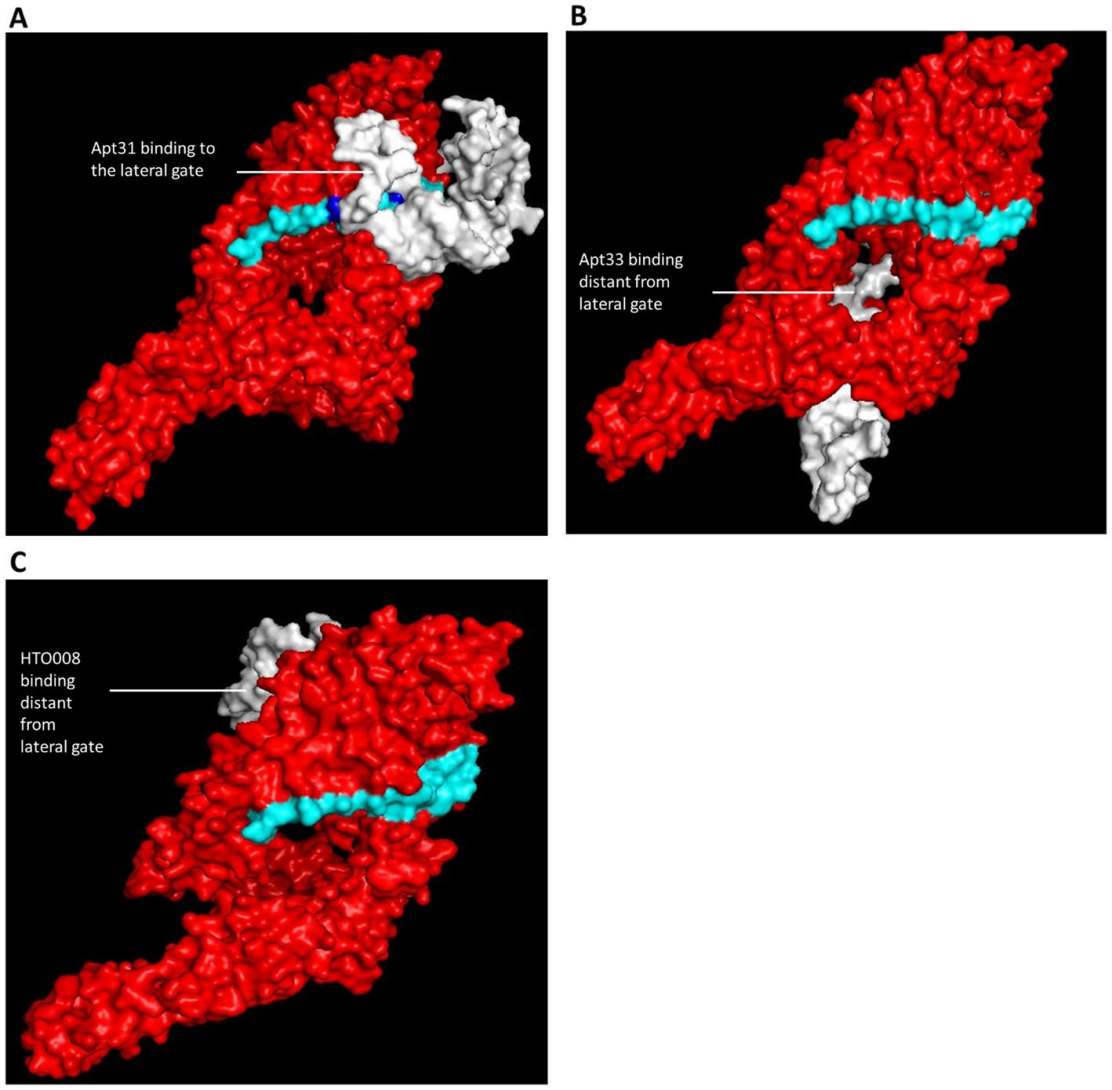
Molecular docking between aptamers and modelled BamA protein. Molecular docking of (**A**) Apt31, (**B**) Apt33 and (**C**) HTO008 (White) to the modelled BamA protein. Apt31 bound to the lateral gate region of BamA, whereas Apt33 and HTO008 bound to the sites distant from the lateral gate. The Darobactin binding residues in the lateral gate that formed non-covalent interactions with Apt31 are highlighted (Blue).

The HADDOCK scores for all the 100 aptamers obtained from ‘Heredia F. DNA/Aptamer’ are shown in the Supplementary Data 1. Out of the 100 docking, eight best aptamer-BamA complexes with the most negative HADDOCK scores were tabulated (Table 1). Complexes that scored less than ‘− 100’ were selected as the best binding aptamers. Negative HADDOCK scores indicate that the complex is energetically stable and the binding is favourable. Apt31 (Daunomycin 10.10v), the top-ranked aptamer in the HADDOCK scores list, was selected for global docking analysis together with Apt33 (Anti-Apple Stem Pitting Virus MT32), which ranked first in the genetic algorithm ranking, and the random oligonucleotide (HTO008). The Gibbs free energy, secondary and tertiary structures of all three aptamers are shown in Table 2. The non-covalent interactions between the aptamer and BamA, such as Van der Waals forces and hydrogen bonds were identified using RING analysis (Table 3). According to Table 3, Apt31 showed the highest interactions where 44% of the aptamer residues interacted with the BamA compared to only 8% of interaction for both Apt33 and HTO008 respectively. The 44% of interaction in Apt31 included interactions between ALA408 in BamA and T11 in Apt31 and VAL410 in BamA and A12 in Apt31 which are similar to Darobactin binding site residues. The residues in the lateral gate region that are involved in both Darobactin and Apt31 binding are highlighted in Fig. 2A. Global docking analysis using the HDOCK server also revealed that Apt31 is binding to the lateral gate region whereas Apt33 and HTO008 are binding distant from the lateral gate (Fig. 2).

**Table 1:** The ranking of best eight aptamers based on HADDOCK score and GA analysis, and their binding at lateral gate.

Aptamer	HADDOCK score	HADDOCK score rank	Euclidean distance	GA rank	Binding at lateral gate
Apt31*	− 130.4 ± 26.4	1	68.103	60	Yes
Apt47	− 125.4 ± 22.7	2	29.061	3	Yes
Apt66	− 123.9 ± 7.5	3	52.157	30	No
Apt62	− 113.4 ± 17.6	4	63.369	50	No
Apt91	− 106.5 ± 5.5	5	47.512	16	No
Apt69	− 106.5 ± 13.1	6	24.273	2	No
Apt33*	− 106.0 ± 4.7	7	19.960	1	No
Apt32	− 104.6 ± 6.1	8	66.443	58	No

*The top ranked aptamers under each ranking.

**Table 2:** Gibbs Free Energy, secondary and tertiary structures of the selected aptamers and negative control.

Aptamer	Gibbs free energy (kcal/mol)	Secondary structure	Tertiary structure*
Apt31	− 0.76		
Apt33	0.78		
HTO008	− 2.77		

*5′ and 3′ of ssDNA are labelled in red and orange respectively.

**Table 3:** RING analysis showing non-covalent interactions between aptamers and BamA protein.

Aptamer	Residue in BamA protein	Residue in aptamer	Type of interaction
Apt31	VAL410	T11	VDW
	VAL410	A12	VDW
	GLN414	A19	VDW
	LEU418	A12	VDW
	ASP475	G16	VDW
	ASP477	G16	VDW
	TYR478	G16	VDW
	TYR478	G17	VDW
	GLU480	G17	HBOND
	LYS642	T18	VDW
	TRP740	T41	VDW
	LEU768	T11	VDW
	PHE773	T9	VDW
	PHE773	G10	VDW
	LEU775	C6	VDW
	ASP783	T41	VDW
	PHE791	G10	VDW
	PHE793	G10	VDW
Apt33	SER405	T5	HBOND
	GLN414	T32	HBOND
	PHE791	T8	VDW
HTO008	PHE412	G4	VDW
	PHE412	C5	VDW
	PHE793	G4	VDW

### Aptamers ranked by genetic algorithm

#### Gaussian distribution modelling of HADDOCK vectors

After the vectors were normalised based on % from 0 to 100%, the potential best aptamers (based on initial classification) were observed to be spread out in their respective range of normalised scores. Ideally, like HADDOCK score, their normalised scores should be clustered together in one Gaussian modal to show some association with good aptamer-BamA binding, as each of the five vectors is supported by literature to have some impact on binding affinity. Hence, as A2 and A5 have multiple clusters of best aptamers with their vector scores more spread out, Gaussian multi modals were calculated for each of the smaller clusters spread out in the range as in Supplementary Table 1.

#### Selection of BamA binding aptamer using GA

To obtain the best distribution of HADDOCK vectors within the 100 aptamers, which range in capability and affinity in binding to BamA, random values of normalised vector scores from 0 to 100% were used as the initial population. The maximum possible normalised scores were obtained after calculations using the Gaussian membership function equation. To explore the potential aptamers capable of binding to BamA, the prediction results of the proposed model were utilised as the initial population of the GA (Supplementary Table 1). By implementing these sequences as the initial population, and the maximum value of each multimodal in A2-A5 vectors, the GA was enabled to learn the best distribution (or best solution in genetic algorithm terms) of A1-A5 vectors in the best aptamer performing in binding to BamA. The best distribution of HADDOCK vectors are as follows: ([8.73009526, 1.91525757, 57.7832437, 36.83345299, 24.81401987]). The fitness value of the best solution is 0.99934/1, which is considered a well-performing GA run, as fitness values of 0–1 are typically close to 0.9. The best fitness value was reached after 499 generations. The GA parameters of which obtained the best solution were num_generations = 500, num_parents_mating = 4, parent_selection_type = “random”, crossover_type = “single point”, mutation_type = “random”.

**Figure 3:**
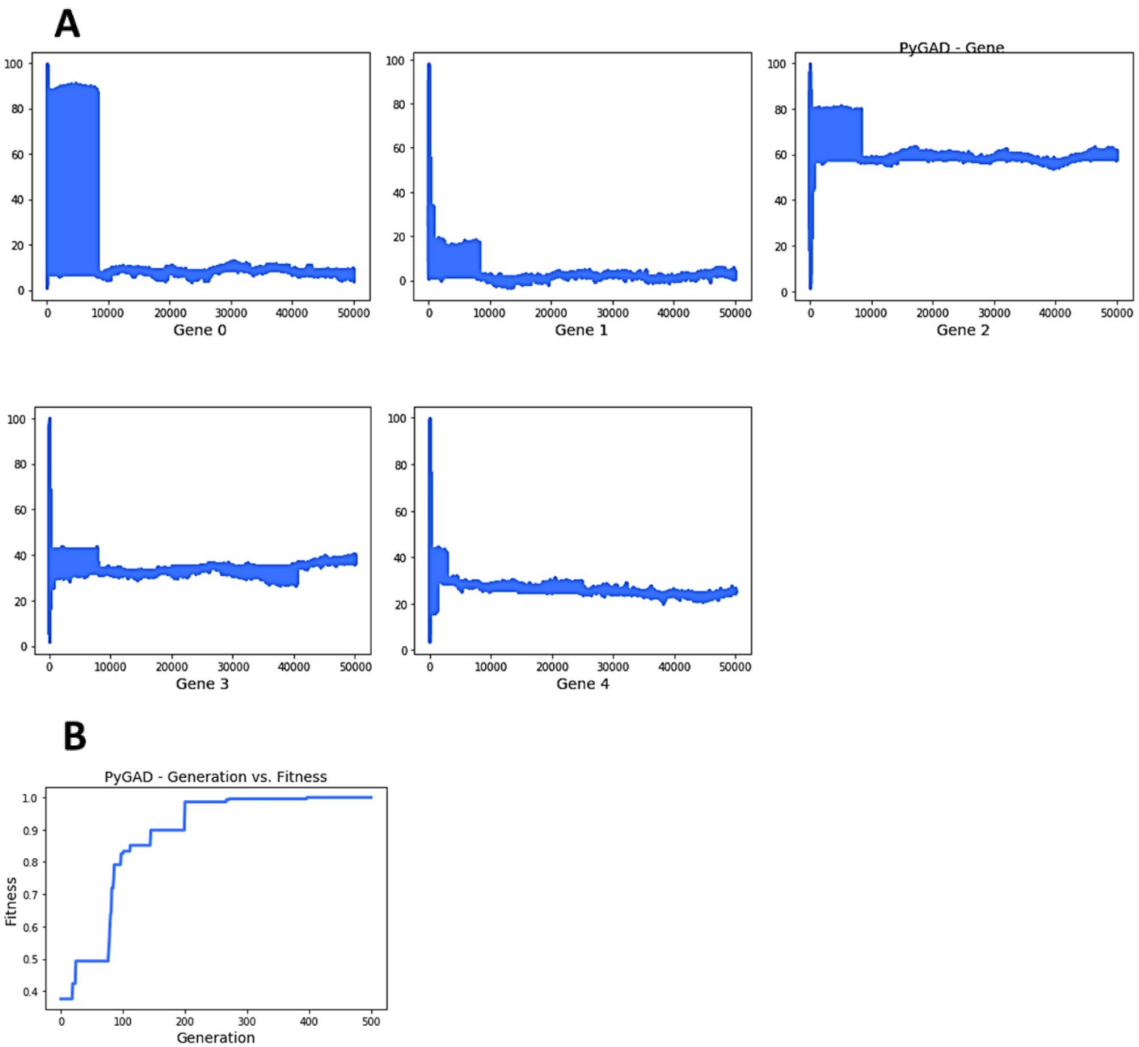
Genetic Algorithm plots of the best distribution. (**A**) Summarises how fitness evolves by generation for each ‘gene’ (in this case the ‘HADDOCK vector’). Genes 0 to 4 refer to HADDOCK vectors A1–A5. (**B**) Summarises how fitness values of solutions change by generations.

In Fig. 3A, it is helpful to know whether a gene value lasts for more generations, as it is an indication of the best value for this gene/HADDOCK vector. For example, the approximate value of 8 for gene 0 (also known as A1) lasted more than 400 generations out of 500. Figure 3B summarises how the fitness values of the solutions change every generation, whereby the fitness value reached 0.9 approximately by generation 150, whilst slowly increasing from 0.90 to 0.99934 from generation 200 to 500. It is worth noting some GA parameters that influenced the efficacy of GA runs, of which efficacy refers to the time taken, a consistent increase in fitness value, and no outlier results (e.g., fitness value beyond 1). They are as follows: when the value assigned to sol_per_pop increases in a range of 50 to 100, fitness values do not go beyond 1 or remain stuck at 0.49), however, as solution per population increases, the GA would require more time to run. Besides, num_generations when kept at 500, the time taken for a full GA run was approximately 4 min, whilst num_generations at 100 takes two minutes. We tested num_generations at 1000 generations to observe how the GA’s potential could be maximised. The 1000 generation run took approximately 20 min and it had weaker fitness results compared to a 500-generation run.

#### Model efficacy analysis

Based on the best distribution of HADDOCK vectors, the Euclidean distance formula ranked all 100 aptamers to observe how close the aptamers were to the ‘best distribution’ (Supplementary Data 1). Overall, Apt33 appears to possess the best distribution of HADDOCK vectors. The ranking based on GA for the aptamer-BamA complexes with the most negative HADDOCK scores are shown in Table 1.

### Effect of aptamers on *P. aeruginosa* survival

**Figure 4:**
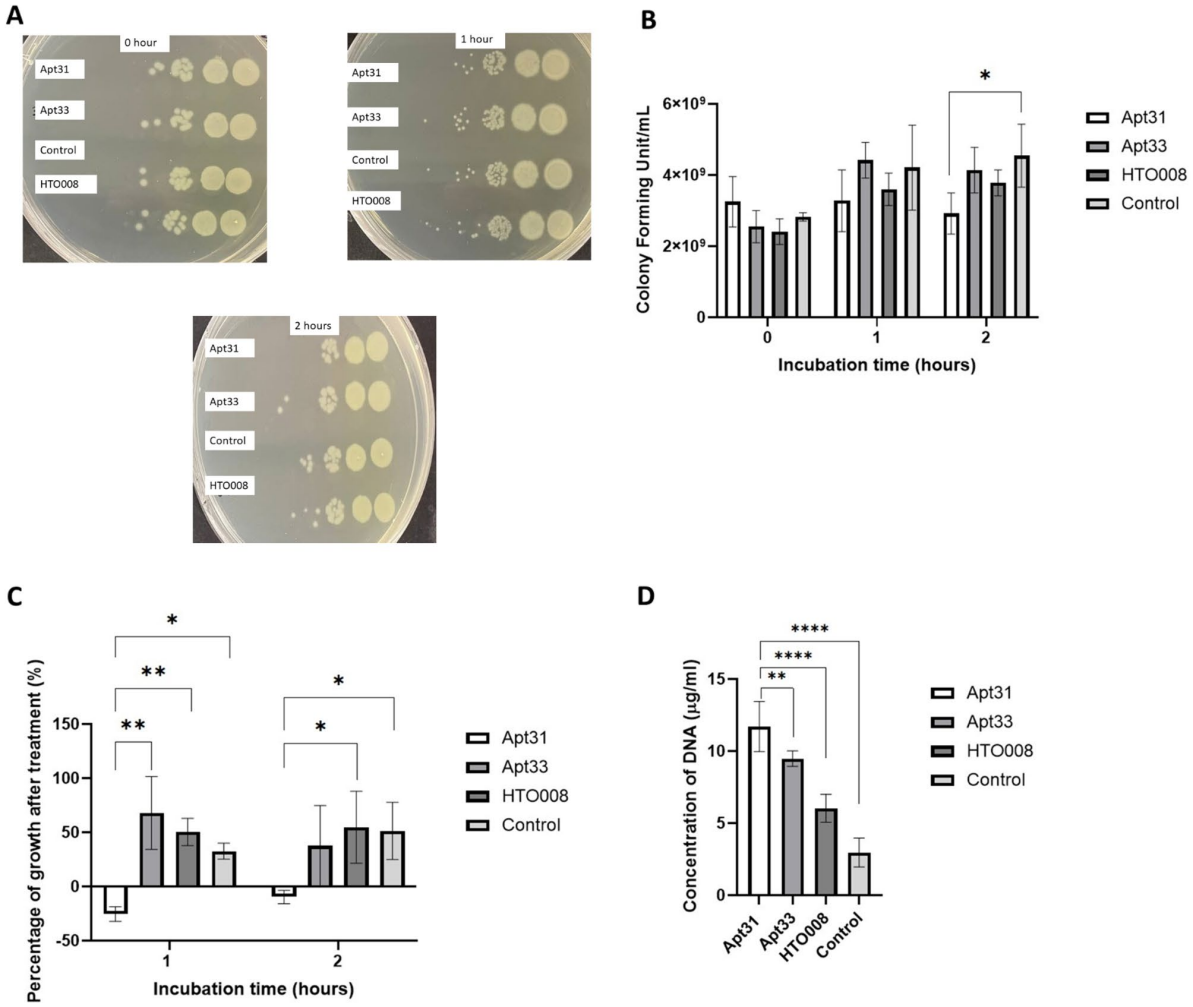
Effect of aptamers on the survival of *P. aeruginosa.* (**A**) Representative images of drop plate method for analysis of antibacterial activity of aptamers on *P. aeruginosa* growth. Each plate represents different time points (0, 1 and 2 h) starting from the dilution 10^4^ (right) to 10^9^ (left). (**B**) Colony forming unit (CFU/mL) of *P. aeruginosa* in the presence and absence of 10 µM of aptamers (n = 5). (**C**) Percentage of *P. aeruginosa* growth after 1- and 2-h incubation with and without aptamers. (**D**) Concentration of DNA released by *P. aeruginosa* after 2 h incubation with 10 μM of aptamers indicating leakage of cytoplasmic materials due to membrane damage (n = 3). Data represent an average of three independent experiments, ± SD shown by error bar; *p value of < 0.05, and **p-value of < 0.01.

Apt31 (top-ranked in HADDOCK scores), Apt33 (top-ranked in GA ranking) and HTO008 (random oligonucleotide) were used for in vitro study to determine the effect of these aptamers on *P. aeruginosa* growth by using a drop plate method (Fig. 4A). As shown in Fig. 4B,C, treatment of *P. aeruginosa* with 10 µM of Apt31 for 2 h showed a significant reduction (p < 0.05) in CFU/mL and percentage of growth. From Fig. 4B, it can be seen that the cfu/ml was increased over time from 0 to 2 h for Apt33, HTO008 and the control group but no effect was observed in the Apt31 treatment group. The negative percentage of growth suggests that there was no growth and at the same time some bacteria cells were dying in the presence of Apt31. It is worth noting that, the inhibitory effects of Apt33 and HTO008 were not as significant (p > 0.05) as Apt31, as these aptamer candidates exhibited higher HADDOCK scores and they were binding distant from the lateral gate in global docking. The measured DNA concentration for the DNA leakage assay was solely dependent on the DNA released by the bacteria cells as purification of the supernatant completely removed the aptamers (Supplementary Fig. 3). In addition to growth inhibition, the concentration of DNA released was also higher for the Apt31 treated group compared to Apt33, HTO008 and the control group (Fig. 4D). This suggests that the Apt31 treatment resulted in compromised membrane integrity in *P. aeruginosa* cells.

## Discussion

Computational screening of potential drugs is gaining interest as it is time-saving and cost effective. In 2010, it was reported that drug discovery costs about USD 1.8 billion and takes about 10–15 years to reach the market^34^, thus computer-aided drug discovery has brought tremendous breakthroughs in the pharmaceutical field over the decades. Besides, as AMR is on the rise, identifying potential targets for Gram-negative species is necessary to overcome this health crisis. BamA is a highly conserved essential outer membrane protein which can be targeted for bacterial growth inhibition^10^. In accordance with WHO, the discovery of a novel antimicrobial agent against *P. aeruginosa* is necessary to treat many nosocomial infections*.* Therefore, aptamers that bind and inhibit BamA activity may emerge as a potential antimicrobial agent.

Although BamA is conserved among Gram-negative species, *P. aeruginosa* showed quite a large genetic distance from other bacteria in the phylogenetic analysis^26,27^. BamA sequence also showed about 82% similarity within the *Pseudomonas* genus, indicating that it is well-conserved among the *Pseudomonas* family. Hence, a universal *Pseudomonas* BamA model is constructed, which is beneficial in designing cost-effective and timesaving antibacterial drugs against various *Pseudomonas* species. The BamA model structured using three closely related templates by Modeller 10v1 showed an RMSD value of 1.781 Å when superimposed with the *E. coli* BamA model from the PDB database (5D0O). This indicates that the *E. coli* BamA model and the *Pseudomonas* BamA model were structurally similar. However, due to the considerable variation in the amino acid sequences between *E. coli* BamA and *Pseudomonas* BamA, the DOPE score profiles of these two structures were slightly different from each other. Nevertheless, the DOPE score profiles were similar between amino acid residues from 360 to 440, suggesting that the β1 strand in the lateral gate region of BamA in *E. coli* and *Pseudomonas* are conserved. Therefore, drugs binding to the lateral gate region in *Pseudomonas* BamA may also be a potential drug that targets the lateral gate in *E. coli* and other Gram-negative species. Other than that, PROCHECK analysis of the modelled BamA shows that 90% of residues fell under the most favoured region, indicating it is a good quality model. Secondary structure prediction by the Ramachandran plot was also compared with existing plot of experimentally determined BamA and it showed that the model consists mostly of β-pleated sheets and a few α-helix which is in agreement with the secondary structure of outer membrane proteins. The similar binding site of Darobactin in both experimental (7P1C) and *Pseudomonas* BamA models further validates the accuracy of the structured model. Darobactin binding site was used as the active site in the HADDOCK 2.4 docking as this antibiotic binds to the lateral gate of BamA and stabilises a closed lateral gate conformation, thereby preventing the entry and exit of nascent outer membrane proteins^13,14^.

Heredia F. DNA/Aptamer database contains all the published unmodified DNA aptamers which are useful in the aptamer repurposing approach^29^. Out of the 100 dockings, only the best aptamer-BamA complexes with the lowest (most negative) HADDOCK score were selected for in vitro analysis. From the HADDOCK 2.4 (local docking) analysis, it can be seen that Apt31 ranked first when it was allowed to bind to the lateral gate region. Additionally, RING analysis showed that Apt31 exhibited about 44% of non-covalent interactions including two interactions at the residues similar to Darobactin binding site. Moreover, the HDOCK server (global docking) also predicted that the best binding pose and position for Apt31 in the BamA protein is the lateral gate region. This explains the reason behind the highest number of non-covalent interactions between BamA and Apt 31. Although Apt33 showed a good negative HADDOCK score by ranking 7th out of the 100 aptamer-BamA complexes, it did not bind around the lateral gate region which suggests that this binding may not affect the BamA activity.

HADDOCK 2.4 analysis consists of various outputs such as HADDOCK score, RMSD from the overall lowest-energy structure, Z-score, Van der Waals energy and Electrostatic energy. Most of the studies only focus on HADDOCK score when studying the protein/drug binding. Although HADDOCK score indicates the best aptamer-BamA complexes by calculating the weighted sum of a variety of energy terms including van der Waals, electrostatic, desolvation, and restraint violation energies, it occasionally does not correspond to the binding affinity of the aptamer to the lateral gate region. Therefore, other features or vectors such as the RMSD and Z-score values should also be considered in the docking analysis to avoid bias. Computer-aided drug designing studies have incorporated GA as part of optimization to identify best-performing drugs^35^. Besides, GA was also used in in vitro SELEX to select high affinity and specificity aptamers^36^. For the first time, we showed that multiple factors should be considered in the HADDOCK docking and this can be done by ranking them using the GA approach. A potential limitation present in this GA analysis, as highlighted by Wirsansky^24^, is that there could be a risk of premature convergence, whereby if fitness in one solution is much higher than the rest of the population in the early generations, it may be duplicated through crossover and eventually cover the whole population in subsequent generations. Hence, the GA could be stuck at a local maximum value prematurely. Lastly, another limitation GA could have is a no-guaranteed solution, as generally GA is utilised to provide decent solutions at a reasonable amount of time^24^.

The antibacterial activity of Apt31, Apt33 and HTO008 were determined by incubating *P. aeruginosa* cells with the aptamers for 1 and 2 h. Although Apt47 showed good ranking in both HADDOCK score and GA analysis in addition to binding to the lateral gate, the very high Gibbs free energy (1.44 kcal/mol) and no well-defined secondary structure predicted for this sequence by UNAFold may suggest that Apt47 may not exhibit good binding in BamA. Hence, Apt47 was excluded from the antibacterial assay. The significant (p < 0.05) reduction in CFU/mL and growth observed in Apt31 treated bacteria cells may be due to the inhibition of BamA activity. The exact mechanism of how Apt31 reduced the survival of *P. aeruginosa* remains unknown. Nevertheless, as both local and global docking showed strong binding of Apt31 to the lateral gate region, Apt31 may block the entry of nascent outer membrane proteins to the lateral gate region and prevent the folding. Accumulation of unfolded outer membrane proteins may lead to cell death^37^ and lack of outer membrane proteins may also affect the outer membrane layer integrity. Loss of membrane integrity results in the leakage of genetic materials such as DNA^38^. Thus, the higher concentration of DNA in the supernatant of Apt31 treated cells indicates that the lack of outer membrane proteins due to BamA inhibition resulted in compromised outer membrane layer integrity and permeability which increased DNA leakage. It is worth noting that, the random oligonucleotide, HTO008 did not show any significant (p > 0.05) effect on *P. aeruginosa* growth and survival as well as in DNA leakage compared to PBS control. Although Apt33 ranked first in the GA analysis, it did not show any significant (p > 0.05) effect on bacterial growth. This may be due to its binding site which was further away from the lateral gate region in the global docking analysis. The GA ranking was based on HADDOCK 2.4 output which uses a local docking approach where the binding site in BamA needs to be specified. Under natural conditions, the Apt33 might bind further away from the lateral gate as implied by the global docking which may not inhibit BamA activity. Moreover, the Gibbs Free Energy for Apt33 is also high which indicates that the folding is unfavourable and the structure has low stability. This may also account for the insignificant effect of Apt33 on *P. aeruginosa* growth. In future, both local and global docking approaches should be analysed concurrently to obtain reliable and specific drug binding. Besides, more advanced machine learning techniques coupled with global and local docking analysis can reveal the best binding aptamers to the lateral gate region in BamA. The antibacterial effect of Apt31 can be further enhanced by making some chemical modifications to the aptamer to increase its binding affinity and stability. The binding of Apt31 to BamA in *P. aeruginosa* with high affinity can be challenging due to the repulsion between the negatively charged aptamer and the negatively charged outer membrane^39^. In addition, nucleases produced by bacteria can also degrade the aptamer, thereby decreasing its antibacterial activity. Hence, modifying the aptamers such as replacing the phosphodiester linkage of DNA with methylphosphonate will reduce the overall negative charge of the aptamers and facilitate the binding of aptamers to the outer membrane protein^40^. Modification to the sugar ring of nucleoside such as 2′-O-methyl-substitution in the aptamer can also resist nuclease degradation and increase the thermostability^40^.

## Conclusion

We showed that the Apt31, an aptamer that binds to Daunomycin, is capable of binding to BamA in *P. aeruginosa* via in silico approach. Local and global docking predicted that Apt31 binds to the lateral gate of BamA with strong interaction. As a proof to this prediction, Apt31 also exhibited a significant (p < 0.05) reduction in *P. aeruginosa* growth. Aptamer repurposing approach saves time, and cost and is less laborious. Incorporating machine learning techniques such as GA in both in silico and in vitro SELEX can increase the accuracy and specificity of selected aptamers. Moreover, targeting a well-conserved protein in bacteria also widens the therapeutic applications among different bacterial groups. Hence, this study could be exploited to cost-effective antibacterial drugs with high therapeutic value in a short period of time.

## Supplementary Information

Supplementary Information 1.

Supplementary Information 2.

## Data Availability

The unmodified DNA aptamer sequences used in this project for docking purposes are available at https://github.com/eipm-uprm/Aptamer-ML and as Heredia F. DNA/Aptamer dataset at https://data.mendeley.com/datasets/76jgjbgndr/1.
