# Supplementary material for: Selecting antibacterial aptamers against the BamA protein in Pseudomonas aeruginosa by incorporating genetic algorithm to optimise computational screening method
Source: Sci Rep. 2023 May 10;13:7582. doi: 10.1038/s41598-023-34643-5 (PMC10170454; doi:10.1038/s41598-023-34643-5)
Supplement: Supplementary file 2 — Supplementary Information 2. [file 41598_2023_34643_MOESM2_ESM.docx]

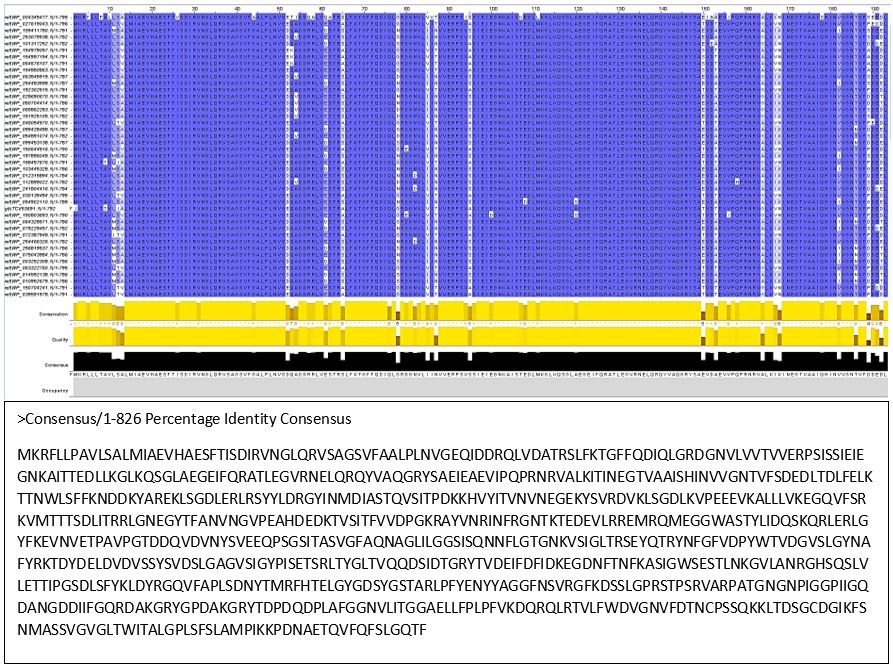


**Supplementary Figure 1:** Multiple Sequence Alignment

Part of multiple sequence alignment showing the first 190 amino acids for the *Pseudomonas* species (purple region indicates the conserved sequence and white region indicates non-conserved sequence) and the consensus sequence from 37 *Pseudomonas* species obtained from COBALT alignment.


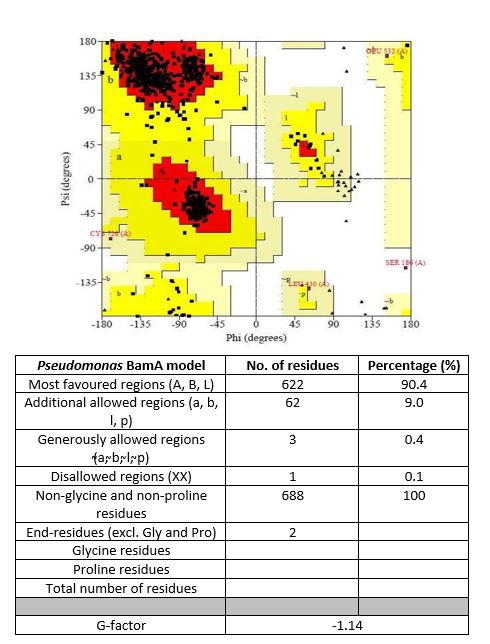


**Supplementary Figure 2:** Quality assessment of the model

PROCHECK analysis of the model with Ramachandran Plot shows that >90% of residues fell under the most favored region and the model consists mainly of β-sheets.


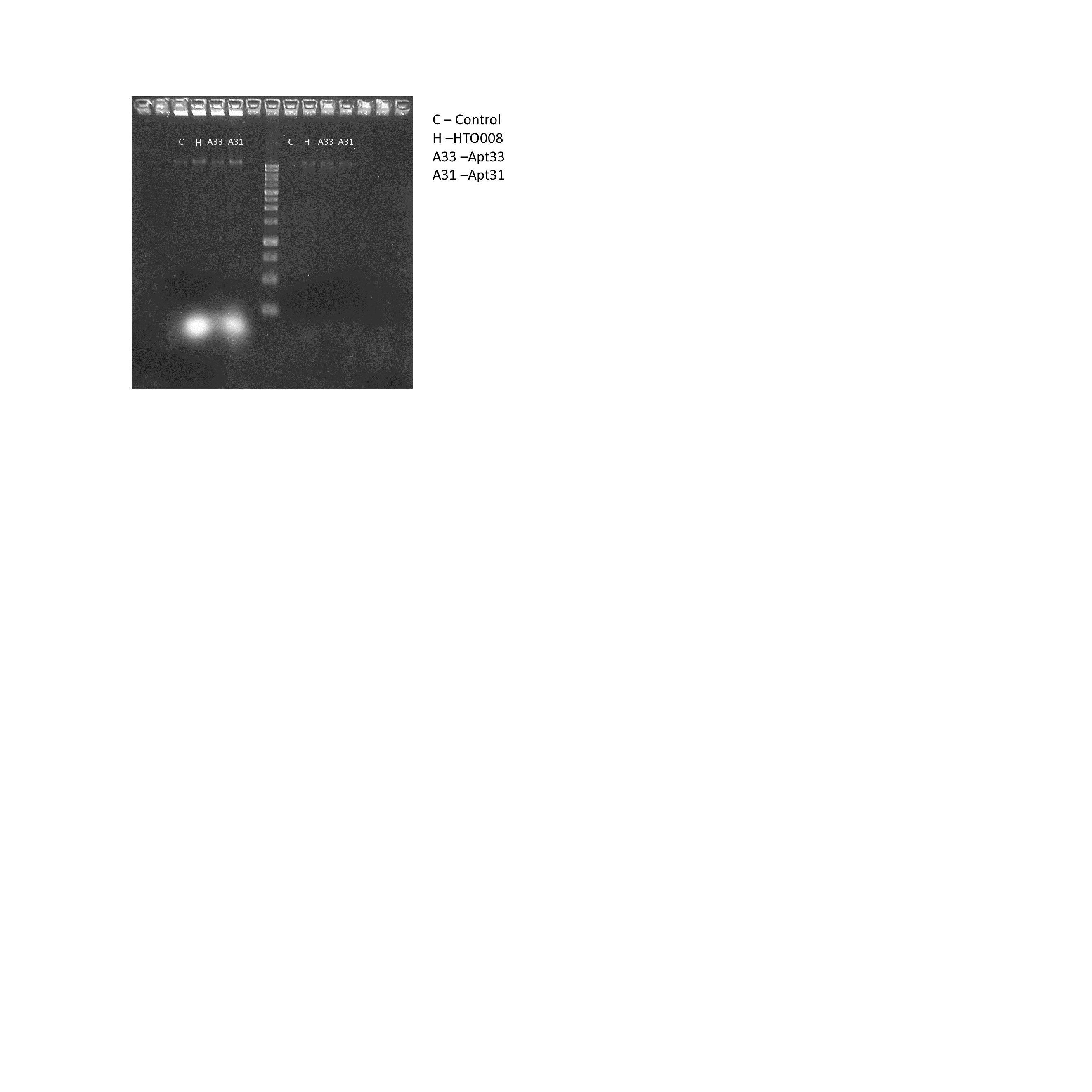


**Supplementary Figure 3:** DNA leakage

Agarose gel showing before (left) and after (right) purification of supernatants collected from treated *P. aeruginosa* cells. Aptamers at around 40 base pairs were completely removed after purification.

**Supplementary Table 1:** Report of each calculated HADDOCK vector with their respective Gaussian models/multimodal, including the mean and standard deviation of the maximum x-value obtained.

| Vector | No. of Gaussian modals/multimodal | function_mean | function_sd |
| --- | --- | --- | --- |
| A1 | 1 modal | [8.67] | [4.038] |
| A2 | 2 multimodals | [1.934, 30.031] | [0.6389, 3.0771] |
| A3 | 4 multimodals | [2.767, 37.862, 50.662, 57.758] | [1.862, 1.025, 0.7493, 1.6173] |
| A4 | 5 multimodals | [46.234, 60.633, 64.455, 73.914, 77.772] | [1.445, 0.336, 0.395, 0.7876, 1.5286] |
| A5 | 2 multimodals | [24.812, 61.184] | [2.378, 2.548] |
